# Assessing Genetic Diversity and Inbreeding Effects in an Endangered Amphibian: Effects of Ecological Restoration and Stochasticity

**DOI:** 10.1002/ece3.73649

**Published:** 2026-05-22

**Authors:** Nadine Nolan, Sarah Stock, Alex Callen, Matt W. Hayward, Sam Wallace, Rose Upton, Michael Mahony, Kaya Klop‐Toker

**Affiliations:** ^1^ Centre for Conservation Science, School of Environmental and Life Sciences University of Newcastle Callaghan New South Wales Australia; ^2^ Centre for African Conservation Ecology Nelson Mandela University Gqeberha South Africa

**Keywords:** amphibians, conservation, drought, population genetics, SNP, waterbodies

## Abstract

The application of population genetic metrics to evaluate the health of a population after undergoing anthropogenic management and experiencing environmental stochasticity remains underexplored in amphibians. Several metrics can be used to measure loss of genetic diversity and decreased gene flow to determine population health, including heterozygosity, inbreeding, and structure. This study assessed the genetic health of the endangered frog, *Rawlinsonia littlejohni* using genetic metrics in a population exposed to human modifications (dam creation) and extreme climatic events (droughts followed by heavy rainfall). We detected strong population structuring, with two distinct sub‐populations located only 2.5 km apart but exhibiting limited gene flow. Genetic differentiation between these sub‐populations, determined by PCoA analysis, decreased after high rainfall in 2021, but returned during drought conditions in 2023, indicating that environmental conditions influence dispersal dynamics. The northern sub‐population, which includes both constructed ponds and natural creek pools, exhibited significantly higher heterozygosity and lower mean kinship. In contrast, the southern sub‐population, dominated by constructed ponds and lacking natural creek connectivity, showed reduced heterozygosity and a marked increase in inbreeding (0.003 in 2020 to 0.162 in 2023). These finding suggest that limited connectivity is constraining gene flow and contributing to reduced genetic health in the southern sub‐population. We propose that additional ponds spaced between sub‐populations may act as stepping‐stones to enhance connectivity and reduce inbreeding. However, our results reveal a key conservation trade‐off: constructed ponds support breeding and population persistence, while natural stream systems facilitate dispersal and genetic connectivity. These findings highlight that restoring long‐term population viability will require multiple, complementary management actions that balance breeding habitat provision with landscape connectivity.

## Graphical abstract

This study evaluated the genetic health of the endangered *Rawlinsonia littlejohni* following habitat modification and climatic variability. We identified two genetically distinct sub‐populations separated by limited gene flow, with heterozygosity remaining higher and inbreeding lower in the northern sub‐population containing both natural and constructed breeding sites. These findings suggest that the availability of natural creek pools supports greater genetic diversity, and that constructing additional ponds between sub‐populations could enhance connectivity and reduce inbreeding.

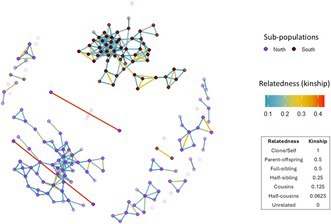

## 1 Introduction

The planet is currently experiencing a significant loss of biodiversity, leading some to consider this the sixth mass extinction event (Johnson et al. 2017; Ward et al. 2024). Amphibians are one of the most affected taxa, a recent report from the second Global Amphibian Assessment (GAA2) increased the number of amphibians on the IUCN Red List from 5725 to 8011 species (IUCN SSC 2023; Luedtke et al. 2023). Current estimates show 40.7% (2873) of amphibian species are globally threatened and categorized as either Critically Endangered, Endangered or Vulnerable (Luedtke et al. 2023). The cause of this global crisis is a combination of threats, including habitat fragmentation and degradation, invasive species, and disease (Blaustein et al. 2018; Luedtke et al. 2023; Nolan et al. 2023). These threats are further exacerbated by climate change and have now been documented across every ecosystem on Earth (Scheffers et al. 2016; Segan et al. 2016; Sopniewski et al. 2022).

Population declines and increased geographical isolation of existing populations often results in a reduction of genetic diversity (Frankham et al. 2019). This loss of genetic diversity is predicted to impact how amphibians respond to declining environmental conditions, such as reduced precipitation, soil moisture and land cover, as small populations with low genetic diversity will have limited potential to adapt to changing conditions (Dolgener et al. 2014; McKee et al. 2017). This interplay between climate change and already reduced genetic diversity creates a feedback loop that further limits adaptive potential. The combined loss of genetic diversity and gene flow within and between isolated populations can decrease individual fitness resulting in lower reproduction and higher mortality rates, which may push populations below the minimum viable size and towards an extinction vortex (Fagan and Holmes 2006; Gilpin 1986; Tanaka 1997). A number of metrics have been used to measure loss of genetic diversity and decreased gene flow to determine population health and individual fitness. This includes measures like heterozygosity, inbreeding, population structure, and kinship. These management‐relevant genetic markers (Leroy et al. 2018) should therefore be a top priority for conservation scientists to maximize long‐term population viability in managed areas (Allentoft and O’Brien 2010; Lacy 1997). Additionally, understanding how genetic diversity changes in response to environmental fluctuations can provide crucial insights for conserving threatened amphibian species.

Many amphibians readily breed in a range of aquatic environments, including both permanent and ephemeral bodies of water (Hunter et al. 2009). The ability of many species to adapt to a wide range of habitats has enabled them to successfully occupy and breed in artificial waterbodies constructed for land management purposes, such as fire control (Brand and Snodgrass 2010; Chester and Robson 2013). While the creation of ponds is designed to meet ecological and land management objectives, their impact on frog dispersal patterns and genetic structure remains uncertain (Covarrubias et al. 2021). Artificial ponds may alter population structure by facilitating movement from natural streams into these human‐made habitats. It is also unclear whether these ponds contribute to low genetic diversity and increased inbreeding in some regions by acting as low‐quality habitats, where individuals may breed but fail to significantly contribute to the overall genetic diversity of the population (Gilroy and Edwards 2017).

**FIGURE 1:**
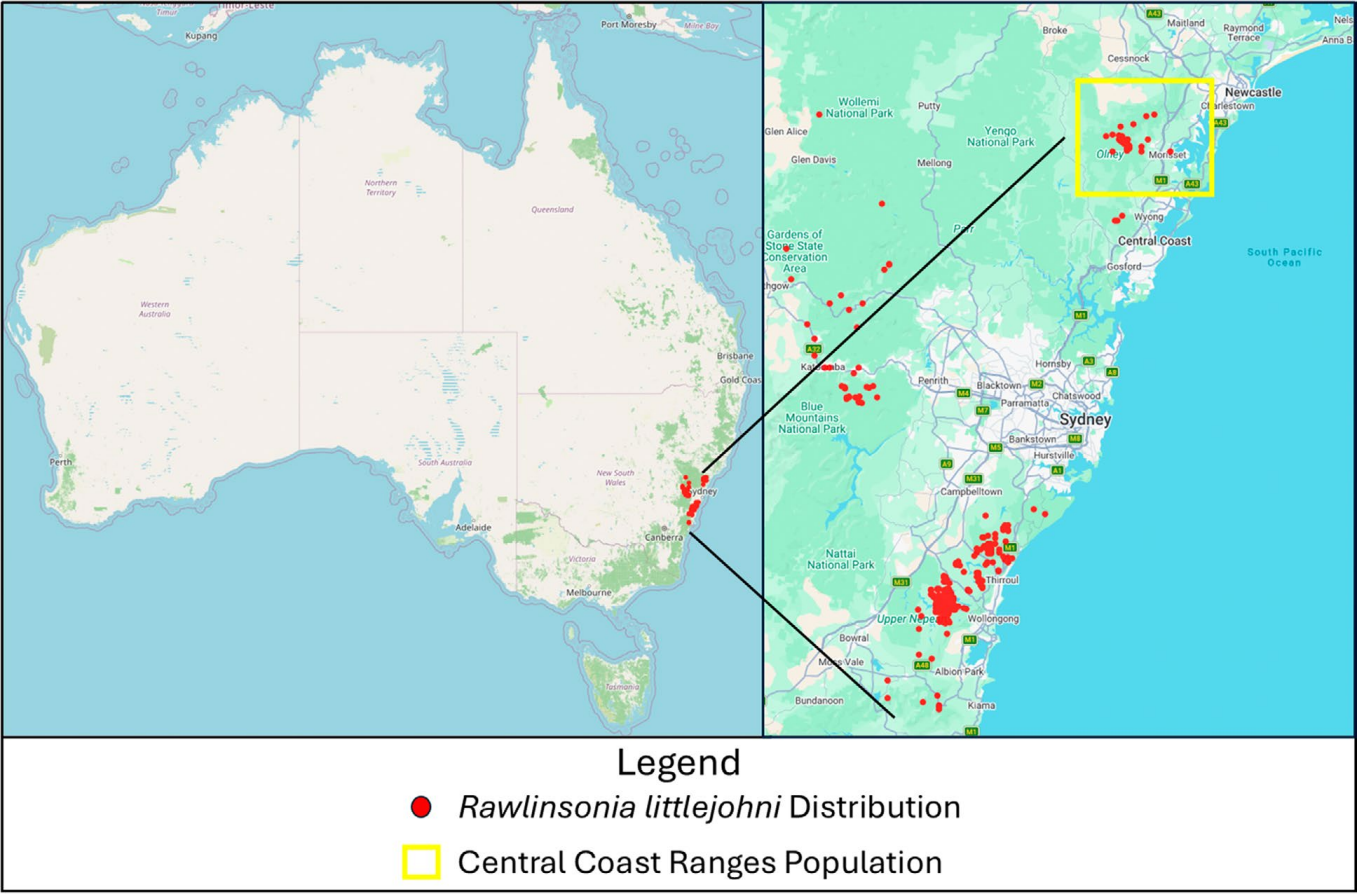
Distribution of the range‐restricted *Rawlinsonia littlejohni*. Black circles represent sightings across the species' range. Yellow rectangle represents the Central Coast Ranges Population (*Rawlinsonia littlejohni* in Atlas of Living Australia Database, 2024).

Littlejohn's tree frog, *Rawlinsonia littlejohni*, is a specialized amphibian endemic to high‐elevation (> 100 m) sandstone plateaus and mountain ranges in central‐eastern New South Wales (NSW), Australia (Figure 1). This species is typically associated with dry sclerophyll forests and heathlands, where it exhibits a strong reliance on permanent or semi‐permanent ponds and slow‐flowing creeks for breeding (Lemckert 2004; Wallace 2023). Breeding occurs predominately in the cooler months (autumn through spring), and larval development is notably slow, often lasting months (Klop‐Toker, Wallace, et al. 2021). Despite these known associations, much of the species' fundamental ecology remains unstudied. There is currently no direct empirical data on individual dispersal distances or fine‐scale movement patterns.

As illustrated in Figure 1, *R. littlejohni* exhibits a highly fragmented distribution along the Great Dividing Range and coastal escarpments of New South Wales. The species' range is currently restricted to five isolated populations, primarily separated by significant landscape barriers such as lower‐elevation valleys and extensive urban development between the Sydney and Illawarra regions (Stock et al. 2023; Mahony et al. 2020). This natural and anthropogenic fragmentation limits connectivity between the surviving high‐elevation ‘islands’ of suitable habitat. Over the past decade, the species has experienced declines, with disease (chytridiomycosis), habitat loss, and natural disasters likely driving declines (Mahony et al. 2020; Klop‐Toker, Wallace, et al. 2021). A previous broad‐scale population genetic examination of the five known and widely separated *R. littlejohni* populations revealed that the northernmost population from the Central Coast Range had low heterozygosity, evidence of inbreeding depression, and a low number of unique alleles (Stock et al. 2023).

The low abundance and cryptic behaviour of *R. littlejohni* makes traditional monitoring methods, such as capture‐mark‐recapture, ineffective, increasing the challenge of measuring population size and movement, and tracking population changes over time (Klop‐Toker, Wallace, et al. 2021; Mahony et al. 2020; Stock et al. 2023). In this study, we aim to demonstrate how genetic monitoring of a population can provide crucial insights into population health estimated using population genetic metrics. We measured population genetics using Single Nucleotide Polymorphisms (SNPs), small genomic variations at a single base position in DNA, to assess (1) population sub‐structuring, (2) kinship (3) genetic similarity between individuals based on geographic distance (spatial autocorrelation at fine scale distance classes, measured in metres to 10s of kilometres) and, (4) heterozygosity, effective population size, and inbreeding, and how each of these measures fluctuated over a 4‐year period. This analysis revealed novel insights into the health of this population and highlighted climatic and landscape variables that may be shaping the structure of this population—findings that have important consequences for the future management and protection of this species.

## 2 Methods

### 2.1 Ethics and Permits

Fieldwork and data collection were conducted in accordance with the Nagoya Protocol on Access to Genetic Resources and the Convention on Biological Diversity (Secretariat of the Convention on Biological Diversity 2011). Relevant permissions were obtained through the New South Wales Department of Planning and Environment under Scientific Licence SL 102642. Animal use and handling protocols were reviewed and approved by the University of Newcastle Animal Care and Ethics Committee (Approval No. A2019‐119).

### 2.2 Surveys and Sample Collection

This study was conducted in the Central Coast Ranges, within the Watagans National Park and Olney State Forest. These areas, elevated 100 metres above sea level, feature a mix of dry sclerophyll forests, eucalypt forests (mountain blue gum *Eucalyptus deanei* , blue‐leaved stringybark *E. agglomerata* ), and drier regions with Sydney peppermint ( *E. piperita* ) and forest oak ( *Allocasuarina torulosa* ) (DECCW 2010). Additionally, sheltered gullies and creek lines in the reserves contain pockets of paperbark and palm forests, as well as warm‐temperate and subtropical rainforest. Geologically, the region consists of sedimentary formations from the Narrabeen Group and Hawkesbury Sandstone, with common rock types such as sandstone, claystone, and conglomerate (DECCW 2010). These geological formations contribute to the region's unique topography, influencing hydrological patterns that are essential for the amphibian habitats. These areas lie at the ecological transition from the moist mid‐north coast forests to the drier Sydney sandstone region.

**FIGURE 2:**
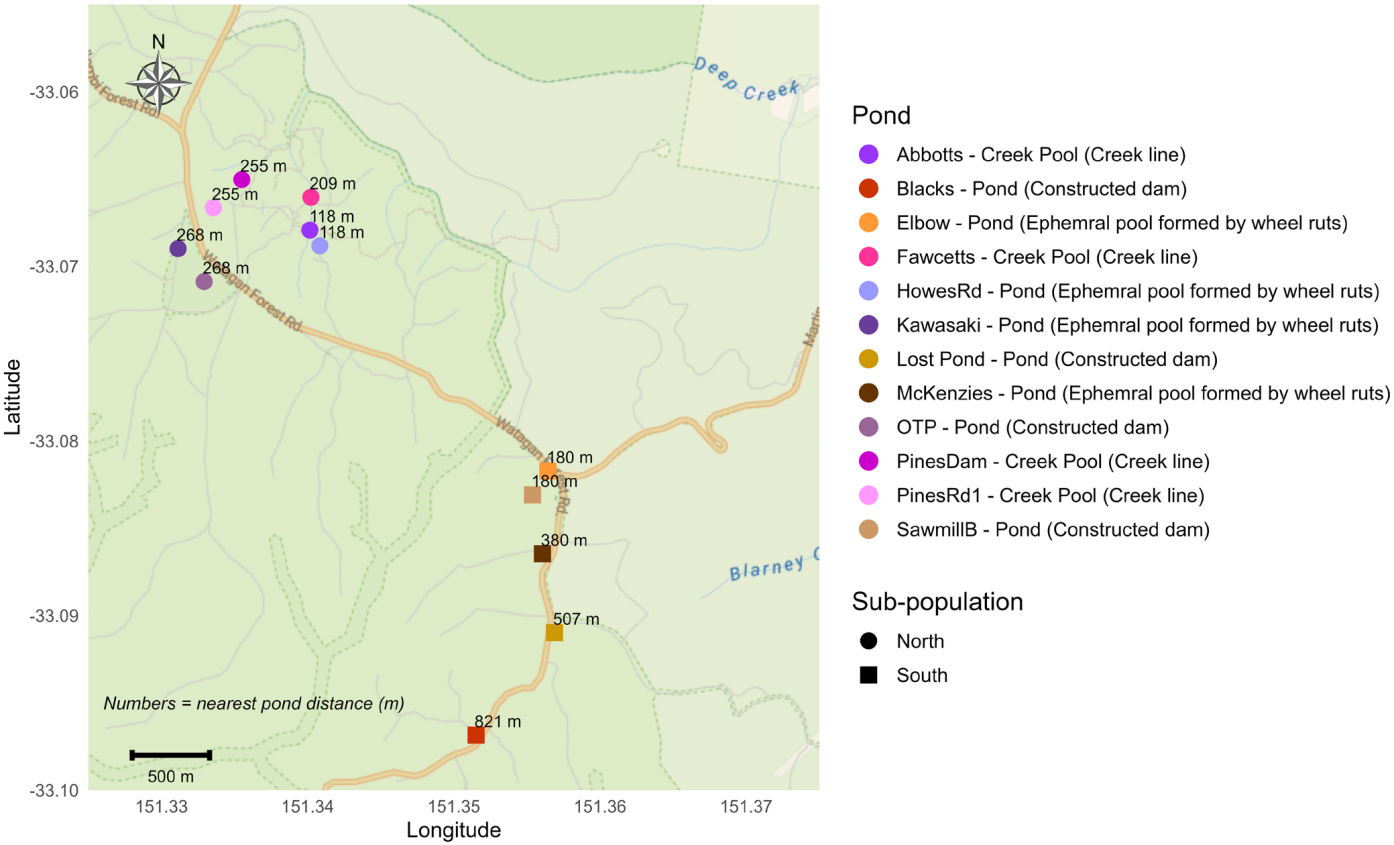
Pond locations in the Central Coast Ranges *R. littlejohni* populations. Circles represent northern ponds and squares represent southern ponds. Grey lines represent roads, with lighter lines indicating smaller trails. Blue lines represent creeks.

Surveys for *R. littlejohni* were conducted between October 2021 and December 2023 in the Central Coast Ranges of NSW (Figures 1 and 2). A total of 12 sites were surveyed across the study period, comprising eight constructed ponds (both ephemeral and permanent) and four creek line pools with low water flow. All sites were selected based on documented historical occupancy, with the exception of two ephemeral ponds discovered in 2022 following heavy rainfall. Because these sites were added in the middle of the study, survey frequency varied across locations; each site was surveyed between 8 and 26 times over the four‐year period, with the lower frequency (eight surveys) reflecting the later inclusion of the two newly discovered ponds *R. littlejohni*. Annual total rainfall and mean maximum temperature data were collected from the Bureau of Meteorology (BoM; http://www.bom.gov.au) monitoring station at Cooranbong (Lake Macquarie AWS), Site Number: 061412 (Latitude: 33.09° S, Longitude: 151.46° E). This station was selected due to its proximity (5 km) to the study area. Data was gathered for the years 2019 through 2023.

Surveys were conducted using a standardized method across all seasons (Summer, Autum, Winter and Spring) and years. Frog surveys involved active Visual Encounter Surveys (VES) (Crump and Scott Jr 1994) of each pond by two to four people after dark, aided by spotlights. Each site visit consisted of two consecutive search rounds: an initial visual search for all frog species, followed immediately by a second targeted search for *R. littlejohni* males through the use of call‐playback. *Rawlinsonia littlejohni* were hand caught and placed into a clean, single‐use plastic bag. After collection, frogs were weighed and measured for snout to‐ vent length (SVL) and right tibia length. Captured *R. littlejohni* were scanned with a hand‐held Trovan microchip reader. If no microchip was detected, indicating a newly captured adult frog, the frog was microchipped using an 8 mm Passive Integrative Transponder (PIT) tag for individual recognition (Christy 1996). Genetic samples were then collected from *R. littlejohni* that were over 40 mm in SVL by taking a 3 mm biopsy from the rear toe webbing. A hand‐held GPS was used to record the latitude and longitude of each pond, and each captured frog was assigned to the corresponding pond based on these coordinates.

Tadpole surveys were conducted during the day and involved the visual inspection and dip‐netting of each pond. Where visibility in a pond was good, tadpoles were counted visually, and abundance recorded as the mean of the counts made by each surveyor present. Tadpoles were identified visually based on their distinct diagnostic features, specifically their jet‐black coloration, blue ventral surface, and characteristic 45‐degree suspension in the water column (Anstis 2017). This distinguishes them from all other sympatric species in the region. When visibility was poor, or there was debris or vegetation in the pond, dip‐netting was used to search for tadpoles. Dip‐netting involved repeated sampling events (approximately ten 2 m sweeps per 25 square metres) where sweeps were made randomly throughout the pond. The number of tadpoles per sweep was recorded. All captured tadpoles were then collected in a container of pond water, and total counts were completed. A maximum subset of ten tadpoles per size cohort per pond were measured for tail and snout‐vent length. A further subset of five tadpoles were biopsied via the collection of a tail tip and a unique number assigned to each tissue sample. Tail tips were only collected from tadpoles longer than 18 mm in total body length and all genetic samples were stored in 70% ethanol.

### 2.3 Climate Variables

Climates variables were collected from the Bureau of Meteorology (BoM), Australian Government. These variables indicate that the Central Coast region experienced drought conditions from 2017 to 2020. Drought is a prolonged, abnormally dry period and is determined in terms of rainfall deficiencies or shortages, compared to average rainfall for the period (BoM). In 2019, the region received only 763.4 mm of annual total rainfall, followed by a significant increase in precipitation in 2020, with 1496.4 mm of annual total rainfall recorded (Table 1). Annual Total rainfall levels remained elevated in 2021 and 2022, at 1343.6 mm and 1894.4 mm respectively, before decreasing again to 907.6 mm in 2023. During the same period, the annual mean maximum temperature fluctuated, peaking at 24.8°C in 2019 and 24.4°C in 2023, with cooler years observed between 2020 and 2022.

**TABLE 1:** Annual rainfall and mean maximum temperature for Central Coast Region (2019–2023).

Climate variables	2019	2020	2021	2022	2023
Annual total rainfall (mm)	763.4	1496.4	1343.6	1894.4	907.6
Annual mean max temperature (°C)	24.8	23.6	23.1	22.8	24.4

*Note:* Data collected from the BoM weather station at Cooranbong (Lake Macquarie AWS): Site number: 061412, latitude: 33.09° S, longitude: 151.46° E.

### 2.4 Genetic Analysis

#### 2.4.1 DNA Sequencing and Initial Filtering

Samples were sent to Diversity Arrays Technology Pty Ltd (Canberra, Australia) (DArT) for DNA extraction. SNP genotyping and discovery was carried out by DArT using their protocol (Kilian et al. 2012). Compared to other sequencing methods, the DartSeq method is able to handle lower DNA input and quality and does not require a reference genome. We followed the methods for DNA sequencing and filtering set out in Stock et al. (2023) and Mahony et al. (2020). We used SNPs as they provide high‐resolution genomic data necessary for accurately detecting fine‐scale population subdivision and determining individual kinship.

The samples collected in this study were co‐analyzed with data collected by Stock et al. (2023) to ensure that the datasets for the Central Coast Ranges could be merged and samples from years 2020 and 2021 could be included in analysis. By merging the two data sets, we were able to examine trends for genetic diversity, effective population size and kinship from 2020 to 2023. Overall, 196 genetic samples (170 adults and 26 tadpoles) were processed. After filtering for double‐sampling and full‐sibling clusters (details below), the final dataset consisted of 189 unique individuals for down‐stream genetic analysis. We included two double‐blind samples from our captive colony consisting of full siblings. This was done to help identify potential double sampling caused by microchip loss and to establish a threshold for detecting related individuals. While PIT tag expulsion occurred, it did not impact the accuracy of our population metrics, as the inclusion of genomic ‘fingerprinting’ for all captures ensured that recaptured individuals were correctly identified.

The DArTSeq sequencing method and filtering pipelines returned a total of 66,712 SNPs (representing 198 total samples), which were further filtered using the *DartR* V2.9.7 package in Rstudio V4.3.2 to ensure the highest quality data (Gruber et al. 2018; Mijangos et al. 2022). We used the function *gl.filter.locmectric* to filter the read depth for both the reference and alternative alleles to > 7. This threshold was selected to minimize sequencing errors while ensuring sufficient coverage for accurate heterozygote calling (Mijangos et al. 2022; Wright et al. 2019). The SNPs were subsequently filtered to fit the following criteria: reproducibility of ≥ 99%; no monomorphic loci; SNP call rate > 97% (SNPs not found in 3% of the individuals); and no secondary SNPs, which were removed by filtering out all but the first SNP with the same CloneID. Finally, we filtered for minor frequency using a threshold of 0.0026 to remove overall rare alleles. Thes filtering thresholds follow standard practices for reduced‐representation SNP datasets (Gruber et al. 2018; Kilian et al. 2012).

Following initial filtering, we evaluated the dataset for the presence of closely related individuals (full siblings/parent‐offspring) and instances of double sampling/recaptures that may have occurred due to microchip loss. Pairwise genetic distances between samples were calculated using the *bitwise.dist* function in *poppr* V2.9.3 package with default settings (Kamvar et al. 2015). Based on double‐blind replicate samples, we established an empirical threshold for genetic distance, whereby pairs differing by less than 0.01% were considered to reflect technical variations (e.g., sequencing or genotyping errors) rather than true biological differences. Individuals falling within this threshold were therefore treated as duplicate samples, and a single representative was retained. In addition, closely related individuals (e.g., full siblings identified be low pairwise genetic distances above the duplicate threshold) were treated as belonging to the same cohort, and one representative individual per cohort was retained to minimize bias in population structure analyses (O'Connell et al. 2019). From the total dataset, five individuals that were within the threshold measures and two full siblings were removed. We refiltered the data thereafter, and further divided this dataset by sample year, creating four smaller subsets for 2020, 2021, 2022 and 2023, which facilitated genetic analysis across different years. Another dataset was created and refiltered (Dataset 2), which retained the two known siblings to be used in the relatedness matrix for thresholding, outlined below.

#### 2.4.2 Genetic Sub‐Structuring

To identify any sub‐structuring within the Central Coast Ranges *R. littlejohni* population, genetic similarity among individuals was visualized. This was achieved using the principal coordinates analysis (PCoA) ordination method, as implemented in the *gl.pcoa* and *gl.pcoa.plot* functions of *DartR*. A scree plot of eigenvalues was used to assess the number of informative PCs to examine, based on the average percentage variation in the original variables explained by the PCs. After viewing the results from the PCoA, it was determined that there was sub‐structuring between the northern and southern pond locations, which are separated by approximately 2.5 km (Figure 2). A PCoA was conducted for each of the four years.

#### 2.4.3 Spatial Autocorrelation

We used spatial autocorrelation analysis to investigate patterns of dispersal and local genetic structure within the Central Coast Ranges population. For this analysis, we excluded all tadpoles to ensure we only assessed individuals of dispersal age, which included 156 individuals. This analysis was conducted using *gl.spatial.autocorr* function from *DartR*, which implements the multivariate spatial autocorrelation method described by Smouse and Peakall (1999). Relationships were quantified using the multivariate genetic correlation coefficient (*r*), which compares a matrix of geographic distances with a matrix of squared genetic distances. We evaluated multiple distance class sizes and selected a 500 m distance class as it represented a biologically relevant scale of dispersal while maintain adequate sample sizes within each class. Analyses were conducted using combined samples from the northern and southern sub‐populations with 1000 permutations and 95% CI. The genetic correlation coefficient (*r*) ranges between −1 and 1, with 0 indicating no genetic correlation at that distance class.

#### 2.4.4 Genetic Differentiation

To assess the degree of genetic differentiation between the northern and southern sub‐populations, we calculated pairwise *F*_ST_ (Weir and Cockerham 1984) using the *gl.fst.pop* function from the *DartR* package, with 1000 bootstrap replications to evaluate whether values were significantly different from zero (Gruber et al. 2018; Mijangos et al. 2022). An *F*_ST_ value of zero designates no difference between sub‐populations, whereas a value of 1 denotes complete differentiation (Luo et al. 2019). To further investigate genetic differentiation between the two sub‐populations within the Central Coast Ranges, we implemented the Bayesian clustering approach in the software STRUCTURE 2.3.4 (Pritchard et al. 2000) on Dataset 1. We tested values of *K* from 1 to 5, running 3 independent replicates per *K*, with a burn‐in of 10,000 iterations followed by 50,000 MCMC iterations to ensure convergence and accurate estimation of ancestry proportions. To determine the most likely number of clusters, we processed STRUCTURE output files using Structure Selector (Li and Liu 2018) and applied the Puechmaille method (Puechmaille 2016), which accounts for uneven sampling by incorporating four estimators: MedMeaK (median of mean assignment probabilities), MaxMeaK (maximum of mean assignment probabilities), MedMedK (median of median assignment probabilities), and MaxMedK (maximum of median assignment probabilities) across sampling locations. We further assessed model fit using the Evanno method (Evanno et al. 2005), which evaluates Δ*K* (Delta *K*)—a second‐order rate of change in Mean LnP(*K*)—to identify the most supported number of clusters. To further refine our results and minimize the risk of overestimating the number of clusters, we conducted multiple replicates using increasing assignment probability thresholds (0.5, 0.6, 0.7 and 0.8).

#### 2.4.5 Genomic Relatedness Matrix (GRN) Analysis

To investigate connectivity and identify potential sub‐structuring within the Central Coast Ranges population, we performed a Genomic Relatedness Network (GRN) analysis. This approach allows for the visualization of fine‐scale genetic structure by representing individuals as nodes and their genetic similarities as connecting lines (Jones and Manseau 2022). While traditional summary statistics can be difficult to interpret when dealing with large datasets, a network‐based visualization can clearly highlight movement patterns and kinship clusters (Allendorf et al. 2010).

To visually assess the relationships of *R. littlejohni* between and within ponds of the Central Coast Ranges, we employed the *gl.grm.network* function from *DartR*, which implements a Genomic Relationship Matrix (GRM) based on the approach by Endelman and Jannink (2012). The GRM the proportion of shared alleles between two individuals by estimating genotype covariance. To visualise these relationships, we applied the *Kamada‐Kawai* (kk) algorithm (Kamada and Kawai 1989), a force‐directed layout that positions nodes based on their genetic distances. We applied a relatedness threshold factor of 0.08 to filter the network for meaningful connections. This qualitative visual representation of kinship was further supplemented by quantitative estimates of kinship and inbreeding using the *Queller and Goodnight (rXY)* and *Lynch and Ritland (rLR)* methods.

#### 2.4.6 Kinship and Inbreeding

To quantify genetic similarity and the extent of parental relatedness within the population, we conducted kinship and inbreeding analysis and tested for spatial and temporal differences using Generalised Least Squar (GLS) regression models. Kinship values represent the degree of relationship between pairs of individuals, while the inbreeding coefficient (*F*) estimates the probability that alleles at a given locus are identical by descent (Frankham et al. 2019).

First, we estimated genetic parameters using moment‐based and regression‐based estimators within the R package *related* V0.8 R (Pew et al. 2015). To ensure comparability with previous studies (e.g., Stock et al. 2023), we used the Queller and Goodnight (1989) *rXY* estimator for pairwise kinship and Lynch and Ritland (1999) *rLR* estimator for individual inbreeding coefficients. These estimates were generated using the *coancestry* function with 1000 bootstrap replicates. Mean kinship values closer to 1 indicate individuals are more related, zero or negative values indicate individuals are unrelated (Manichaikul et al. 2010). Kinship determined from the *Quellergt* estimator were averaged to get mean kinship scores for within the Central Coast Ranges sub‐populations.

All subsequent statistical comparisons of these values were performed using GLS models via the *gls* function from the *nlme* package (Pinheiro and Bates 2024). We chose GLS as it allows for the modelling of heteroscedasticity and non‐independent error structures often found in population genetic data. Specifically, we assessed differences in mean inbreeding (*F*) between the two sub‐populations and among sampling years, including an interaction effect between sub‐population and year. Significant effects were further investigated using post‐hoc pairwise comparison using the function *pairs* was employed.

#### 2.4.7 Genetic Diversity

From the SNP data, observed heterozygosity, expected heterozygosity, unbiased expected heterozygosity, and *F*_IS_ were calculated using the *gl.report.heterozygosity* function from the *DartR* package. The standard deviation for *F*_IS_ was calculated using *boot.ppfis* from the *hierfstat* V0.5‐10 package with 100 bootstraps (Goudet 2005). Observed heterozygosity, expected heterozygosity, unbiased expected heterozygosity, and *F*_IS_ were determined across years. The SNP data used to determine initial genetic diversity was split into four years (2020, 2021, 2022 and 2023) based on sample collection date and re‐analyzed using the *gl.report.heterozygosity* function from the *DartR* package. *F*_IS_ values detect deviations from random mating in recent generations. An *F*_IS_ of zero indicates a level of inbreeding equal to the expected value based on allele frequencies in the population, a positive *F*_IS_ indicates inbreeding; a negative F indicates avoidance of inbreeding (Nichols 2017).

In addition, we calculated autosomal heterozygosity, which includes invariant (monomorphic) sites. To calculate autosomal heterozygosity, we applied different filtering to the SNP data, which contained 175 samples and 18,190 loci. This process involved filtering loci and individuals with a call rate threshold of < 0.70 and < 0.90, respectively, and secondaries were not removed (i.e., multiple SNPs on the same read). We then estimated the total number of invariant sites by using the *gl.report.secondaries* function. All other filtering steps were the same as initial filtering performed above in Section 2.4.1. We used the *gl.report.heterogysity* function from the *DartR* package to obtain autosomal heterozygosity (Mijangos et al. 2022). Significant differences in mean autosomal heterozygosity between the sub‐populations and years were assessed using a Generalized Least Square (GLS) regression model in R using the *gls* function from the *nlme* package (Pinheiro and Bates 2024). To detect significant autosomal heterozygosity values between sub‐population and years, a pairwise comparison using the function *pairs* was employed.

#### 2.4.8 Effective Population Size

We estimated the effective population size (*N*_e_) for the norther and southern sub‐populations using the software NeEstimator V2.1 (Do et al. 2014). This analysis was conducted using the Linkage Disequilibrium (LD) method (Waples 2006; Waples and Do 2008), which uses the non‐random association of alleles at different loci to infer the number of breeding individuals. We assumed a random mating model and applied a critical allele frequency threshold of 0.05. This threshold was used to exclude rare alleles that may bias *N*_e_ estimates, ensuring that the results reflect the core genetic diversity of the sub‐populations.

## 3 Results

### 3.1 Survey Results

We conducted 304 pond surveys across 12 ponds and captured a total of 208 *Litoria littlejohni* individuals, of which 196 were tissue sampled for SNP genotyping. A total of 17 recapture events were recorded between 2020 and 2023, identified through either microchips (12 individuals) or genetic analysis (five individuals).

Recapture data showed that two frogs were recaptured twice in 2020, one frog was recaptured twice in 2021, one frog was first captured in 2021 and recaptured in 2022, and another frog was first captured in 2021 and recaptured in 2023. All five individuals identified as recaptures through genetic analysis were found at the same pond where they were originally captured. Among the microchipped frogs, seven were recaptured in 2022—four at the same pond and three at different ponds. In 2023, six additional frogs were recaptured, with one found at a different pond. The observed movement between ponds, recorded through microchips, ranged from 170 to 680 m (Table S1).

The low recapture rate highlights the cryptic nature of *R. littlejohni*, reinforcing previous suggestions that a substantial portion of the population remains undetected in mark‐recapture studies. Given these limitations, estimating population size using traditional mark‐recapture methods alone is challenging. Instead, genetic approaches, such as estimating effective population size (Ne) through SNP‐based analyses, provide a more reliable method for assessing population size and genetic diversity in this species.

### 3.2 Genetic Sub‐Structuring

**FIGURE 3:**
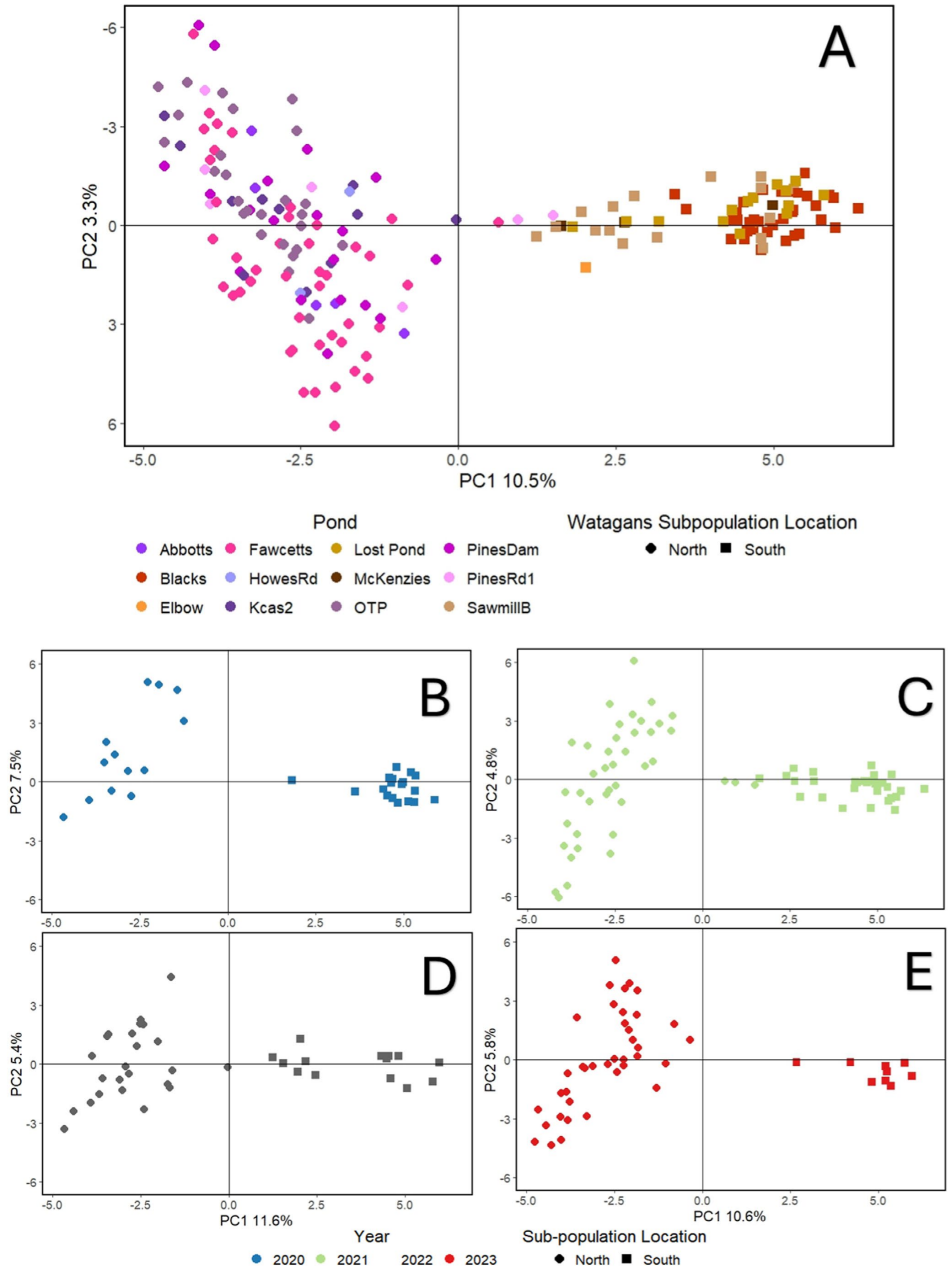
Principal Coordinate Analysis (PCoA) for *R. littlejohni* SNPs collected from the Central Coast Ranges. (A) PCoA plot based on 4326 SNPs collected over 4 years, (B) PCoA plot based on 193 SNPs collected in 2020, (C) PCA plot based on 1983 SNPs collected in 2021, (D) PCoA plot based on 1781 SNPs collected in 2022, (E) PCoA plot based on 1588 SNPs collected in 2023.

After initial filtering and removal of duplicate samples and closely related individuals, we retained 2020 polymorphic SNP loci scored from 186 individuals of *R. littlejohni*, including both adults and larvae (Dataset 1). Genetic sub‐structuring was assesses using principal coordinate analysis (PCoA) based on pairwise genetic distances derived from SNP data. In the initial analysis including all samples across years, the proportion of genetic variance explained by the first two principal coordinate axes were 10.5% and 3.5% respectively. Although the variance explained by individual axes was relatively low, this is typical for high‐dimensional SNP datasets and does not preclude the detection of biologically meaningful structure. Two genetic sub‐populations are apparent and describe geographically discrete northern and southern sub‐populations (Figure 3A).

A total of 193, 1983, 1781 and 1588 polymorphic SNP loci were scored from 31, 70, 39 and 47 individuals in the years 2020, 2021, 2022 and 2023, respectively. In 2020, the first PC axis accounted for 12.9% of the genetic variance, while the second axis explained 7.5% (Figure 3B). In 2021, these values decreased to 11.5% and 4.8% (Figure 3C). A similar trend was observed in 2022, with the first and second axes explaining 11.6% and 5.4% of the genetic variance (Figure 3D). By 2023, the first axis explained 10.6% of the genetic variance and the second axis 5.8%, suggesting a further slight decrease in the genetic variance between the two sub‐populations (Figure 3E). These differences are relatively small and may reflect variation in sample size and the number of loci retained across years rather than substantial biological change. Notably, the overall pattern of genetic sub‐structuring between the northern and southern groups remained consistent across all years. While sample sizes differed among years, all exceeded commonly accepted thresholds for detecting genetic structure, and the consistency of clustering across years supports the robustness of the observed pattern.

### 3.3 Spatial Autocorrelation

**FIGURE 4:**
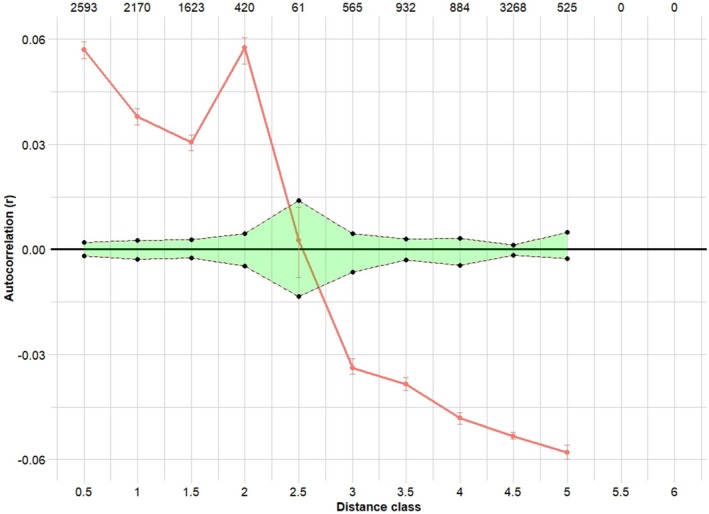
Spatial autocorrelation correlogram of the entire sample for Central Coast Ranges *L. littlejohni* populations. The autocorrelation coefficient (*r*) represents the genetic similarity among individuals within each distance class, calculated from pairwise SNP‐based genetic distances. Distance classes are shown in kilometres, with a bin width of 500 m. Black points represent observed *r* values for each distance class. Two green dotted lines indicate the 95% confidence interval under the null hypothesis of a random spatial distribution of *R. littlejohni*, generated by permutation. The red points and error bars represent bootstrap estimates of *r* and their associated 95% confidence interval, indicating the variability and uncertainty around observed *r* values across resampled datasets. Points are connected to illustrate the trend in genetic autocorrelation across increasing distance classes.

The correlation coefficient (*r*) in the spatial autocorrelation analysis was significantly positive for individuals at 0.5, 1, 1.5 and 2 km apart, where *r* values exceeded the upper 95% confidence interval generated under permutation of individuals among distance classes, indicating high site fidelity at these class distances (Figure 4). There was no correlation at 2.5 km. At distances greater than 2.5 km, *r* values fell below the lower 95% confidence interval, indicating significantly negative spatial autocorrelation, suggesting that there is some factor impacting connectivity and limiting movement and dispersal.

### 3.4 Genetic Differentiation

The pairwise *F*_ST_ value between the northern and southern sub‐population of the Central Coast Ranges indicates some genetic differentiation (Table 2), with the comparison being significantly different from zero. *F*_ST_ values range from 0 to 1, with higher values indicating a significant degree of differentiation among populations. The highest pairwise *F*_ST_ value between the two sub‐populations was observed in 2023. STRUCTURE analysis using the Evanno method suggested the presence of two genetic clusters (*K* = 2; Figures S3 and S4), which may indicate some level of genetic structuring within the population. However, despite *F*_ST_ values indicating some genetic differentiation, all four Puechmaille estimators consistently identified a single optimal cluster (*K* = 1; Figure S2).

**TABLE 2:** Pairwise *F*_ST_ values between north and south sub‐population of the Central Coast Ranges *R. littlejohni* population.

	Comparison	North
*F*_ST_ all years	South	0.098*
*F*_ST_ 2020	South	0.084*
*F*_ST_ 2021	South	0.097*
*F*_ST_ 2022	South	0.087*
*F*_ST_ 2023	South	0.122*

*Note:* *F*_ST_ is a measure of genetic differentiation between populations, ranging from 0 (no differentiation) to 1 (complete differentiation).

* Indicates signifcant values.

### 3.5 Relatedness

**FIGURE 5:**
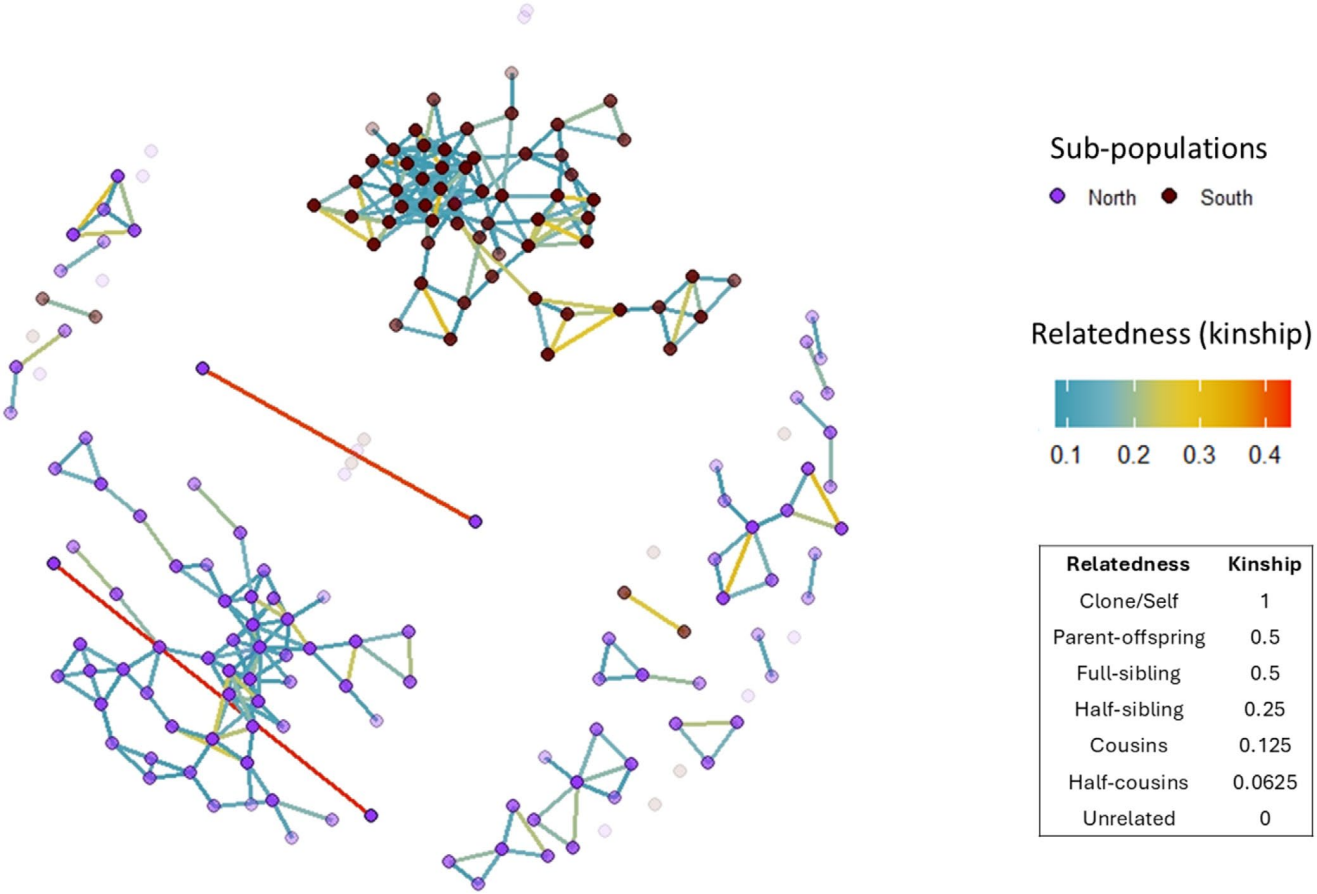
Genomic relationship matrix (GRM) based on genetic samples collected from *R. littlejohni* across 4 years within the Central Coast Ranges population, including two known sibling pairs. Each node represents an individual frog and connecting lines indicate related individuals. Nodes are colour‐coded by sub‐population (north and south) and lines' colours represent level of relatedness (kinship) calculated using the *gl.grm* function. Kinship values range from 0 to 1, where 1 represents identical individuals (or twins) and 0 represents unrelated individuals, with intermediate values indicating varying degrees of relatedness. Node transparency reflects the number of connections (relationships) for each individual, with more opaque nodes indicating a higher number of related connections.

Relatedness values in the genomic relationship matrix (Figure 5) show a clear divide between individuals sampled from populations in the north and south. Individuals from the southern ponds show no connection by kinship to the northern ponds, however in the Genomic relationship matrix (Figure 5), there are two pairs from the southern populations sitting within the northern sub‐population. In the northern sub‐population, the genomic relationship matrix revealed one primary cluster alongside 14 smaller, distinct clusters and six individuals that were not associated with any cluster. In the southern sub‐population, there are only three clusters, but the majority of individuals are concentrated in a single cluster, and only five individuals not connected to others. In the southern sub‐population, high levels of kinship occur between individuals from Blacks Pond, Lost Pond, and Sawmill B Pond, observed in the top centre of the genomic relationship matrix (Figure S1). In the northern sub‐population, high levels of kinship occur between Pines Dam, OTP, Fawcett, and Pines Rd1 (Figure S1). The two sets of known siblings included in this analysis are represented in the genomic relationship matrix by two red lines connecting two siblings from Kcas2 on the centre left and two siblings from OTP on the bottom left. The two set of siblings were used to determine the threshold for the genomic relationship matrix relatedness values.

### 3.6 Effective Population Size and Mean Kinship

#### 3.6.1 Northern Sub‐Population

**FIGURE 6:**
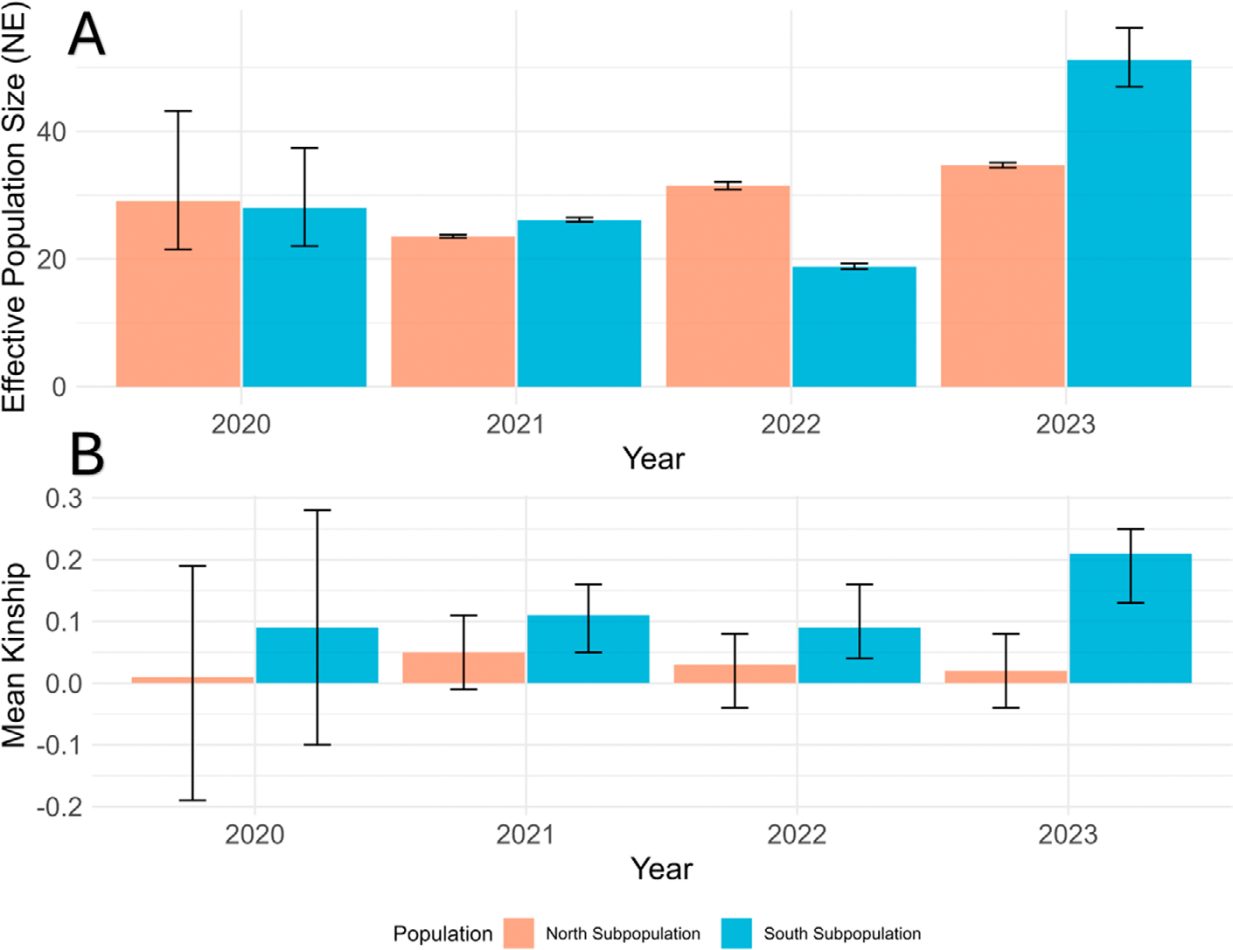
Mean effective population size (A) and mean kinship (B) for each year between the northern and southern sub‐populations for *R. littlejohni* in the Central Coast Ranges.

The effective population size of the northern sub‐population dropped from 28 to 23 effective individuals between 2020 and 2021, followed by a steady increase over the subsequent two years, reaching 35_[34.3,35.1]_ individuals in 2023 (Figure 6A). Mean kinship was consistently low across the four years with a slight increase observed in 2021(see Table S2).

#### 3.6.2 Southern Sub‐Population

The effective population size in the southern sub‐population dropped from 27 individual in 2020 to 19 individuals in 2022, then increased to 51 individuals in 2023. Mean kinship was higher in the southern sub‐population compared to the northern sub‐population across all years, where it remained constant from 2020 to 2022. However, in 2023, mean kinship more than doubled, increasing from 0.09 in 2022 to 0.21 in 2023 (Figure 6B).

### 3.7 Autosomal Heterozygosity and Inbreeding

Mean autosomal heterozygosity was significantly higher in the northern sub‐population (0.063) compared to the southern sub‐population (0.061) (Generalized Least Squares regression, *t* = −2.3, df = 180, SE = 0.0008, *p* = 0.02) (Figure 6A). A Pairwise comparison showed autosomal heterozygosity was significantly lower in 2021 (0.0611) compared to 2020 (0.0649) (*t*= 3.27, SE = 0.0011, *p* = 0.006) and 2022 (0.0643) (*t*= −2.98, SE = 0.001, *p* = 0.017) in both sub‐populations (Table S3). There was no significant interaction effect for autosomal heterozygosity between years and sub‐population.

**FIGURE 7:**
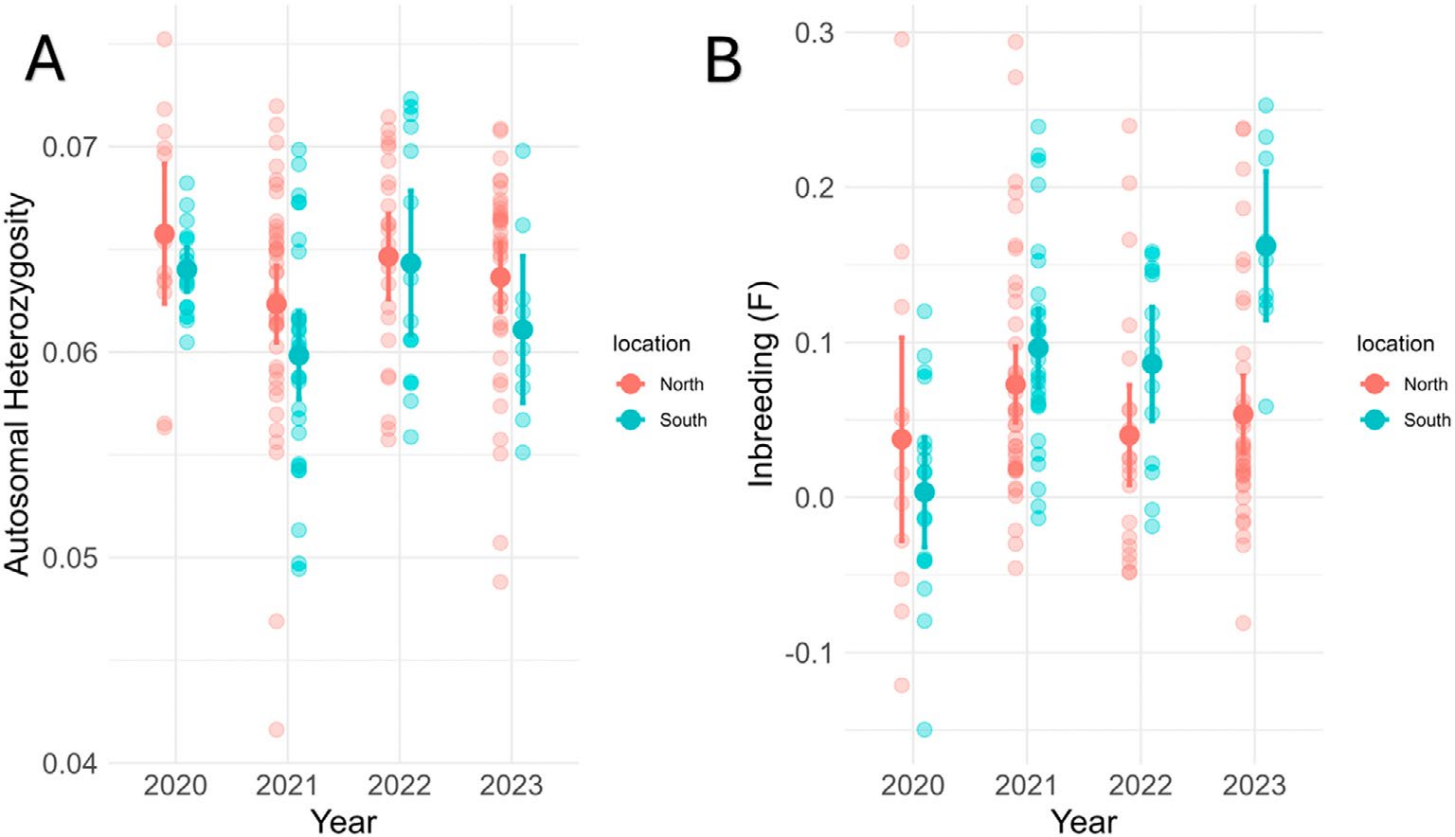
Mean autosomal heterozygosity (A) and inbreeding coefficient (B) for each year between the northern and southern sub‐populations for *R. littlejohni* in the Central Coast Ranges.

There was no significant change in inbreeding (*F*) in the northern sub‐population across years. Inbreeding in the southern sub‐population increased significantly from year 2020 (0.003 *F*) to 2023 (0.162 *F*) (Generalized Least Squares regression: *t* = −5.108, df = 177, SE = 0.031, *p* =< 0.0001), Figure 7B. A significant difference in inbreeding was seen across all years in northern sub‐population compared to year 2023 in the southern sub‐population (Table S4). Positive *F* scores between 0 and 0.05 are considered low, scores between 0.06 and 0.14 deemed average (Hartl and Clark 1997; Wright 1965).

## 4 Discussion

We analyzed population genetic metrics to monitor the genetic health of a small, isolated population of an endangered amphibian in response to habitat creation and environmental stochasticity. Baseline genetic analysis using SNPs in 2020 demonstrated that the *R. littlejohni* population had low genetic diversity and high inbreeding coefficients. Our analysis of genetic samples collected for three consecutive years following initial work by Stock et al. (2023) revealed clear population sub‐structuring between northern and southern sub‐populations with limited gene flow. Drought conditions appeared to affect the dispersal ability of *R. littlejohni*, with increased structure observed during dryer years. In comparison, an increase in dispersal was observed during heavy rainfall events that created new ephemeral ponds across the landscape in years 2021 and 2022. In these years, our Principal Coordinate Analysis revealed decreased structure, likely reflecting greater movement of individuals facilitated by environmental processes such as rainfall and surface water availability. In the northern sub‐population, where breeding habitats included a mixture of constructed dams and natural creek pools, we found higher heterozygosity and lower mean kinship. In contrast, the southern sub‐population, dominated by constructed dams, exhibited lower heterozygosity and higher mean kinship levels. These patterns indicate reduced genetic diversity and connectivity in the southern sub‐population, suggesting increased vulnerability to ongoing environmental and demographic pressures. These findings highlight the importance of breeding habitat type and connectivity in facilitating dispersal and maintaining genetic diversity, which are critical for long‐term population resilience. Founder effects, where new populations are established by a small number of individuals leading to reduced genetic variation, may also contribute to the observed genetic patterns (Jamieson 2011; Haileselasie et al. 2018).

### 4.1 Movement and Population Sub‐Structuring

Previous genetic work indicated there was a single *R. littlejohni* population in the Central Coast Ranges (Stock et al. 2023), however, our analyses detected genetic sub‐structuring, with two distinct sub‐populations representing a geographical north‐south split. Despite being separated by just 2.5 km, the northern and southern sections of the Central Coast populations exhibited an *F*_ST_ value of 0.098, indicating moderate genetic differentiation between the sub‐populations. The distance between these sub‐populations is shorter than the 6 km separation observed among the three populations on the Woronora Plateau, that show less genetic differentiation, as identified by *F*_ST_ values ranging from 0.071 to 0.085 (Stock et al. 2023). The genetic isolation observed between the two sub‐populations in the Central Coast Range suggest that *R. littlejohni* breeding habitat is highly fragmented in this area and that dispersal between breeding ponds by *R. littlejohni* is rarely successful. Habitat fragmentation can restrict movement, even in species that are physically capable of dispersing over significant distances. This may be the case for the Central Coast Ranges population, given the observed dispersal patterns in the Cordeaux population in southern NSW, which occurs in a relatively continuous and less fragments habitat and shows no genetic sub‐structuring, functioning as a single population (Stock 2023). Similar patterns have been observed in other *Litoria* species, where habitat fragmentation has led to fine‐scale genetic structuring and recent population declines, as seen in the Kuranda Treefrog ( *Litoria myola* ) (Bertola et al. 2023). These findings highlight the importance of maintaining habitat connectivity to support gene flow and population stability.

Stream systems are a crucial component of *R. littlejohni*'s breeding habitat, with the species favouring first or second‐order, slow‐moving streams flowing over sandstone substrates (Wallace 2023). The most genetically diverse populations are found in areas with well‐connected stream networks, such as those found on the Woronora Plateau, which likely facilitate movement between breeding sites (Stock et al. 2023). In the Central Coast landscape, the lack of connectivity of aquatic habitat between slow‐moving streams over a sandstone geology may restrict connectivity between the two sub‐populations. The northern and southern sub‐populations occur on a narrow plateau and are not connected by any creek lines. Therefore, the reduced movement between these sub‐populations may be caused by the frog's inability to disperse through dry, ridge‐line habitat (Figure 2). Additionally, Stock et al. (2023) demonstrated that high rainfall can wash tadpoles downstream along creek‐lines, thereby facilitating dispersal at different life stages. In the Central Coast Ranges, creek lines flow in opposite directions from ridge tops—Wyong River flowing south and Dora Creek flowing north—draining into distinct catchments. This hydrological separation makes it unlikely for tadpoles to be washed between sub‐populations, potentially contributing to the strong sub‐structuring observed in the region (Lemckert 2004; Wallace 2023).

The creek lines are established habitat for the northern sub‐population of *R. littlejohni* in the Central Coast Ranges and thus play a crucial role in maintaining genetic health. Heterozygosity was significantly higher in the northern sub‐population compared to the southern one, likely due to the presence of both constructed ponds and natural pools along the creek system, which served as breeding habitat. The close proximity of these habitats (< 1 km) likely enhances dispersal opportunities, facilitating genetic mixing and reducing the chances of related individuals breeding, arresting the potential for further genetic erosion in this sub‐population. Conversely, in the southern sub‐population, where artificial ponds constitute the majority of breeding habitat, we observed higher levels of kinship and mean kinship, along with reduced heterozygosity. This study provides novel evidence that pond creation may influence the genetic structure of amphibian populations. Given the proximity of the ponds within this landscape (all within 4 km), we assumed that the population would exhibit a panmictic structure (random mating throughout), but instead, we observed higher genetic structuring than anticipated. This may be due to the discrete nature of the ponds, which could be limiting movement between breeding sites compared to what would naturally occur in a continuous stream system.

Similar patterns have been observed in other *Litoria* species, where dispersal and genetic structure are strongly influenced by landscape features. For example, *Litoria nannotis* and *Litoria serrata* exhibit increased dispersal and reduced genetic structuring in wetter environments, while more fragmented upland–lowland systems are associated with reduced movement and increased structure (McKnight et al. 2019; Rowley and Alford 2007). Similarly, the terrestrial‐breeding frog *Pseudophryne guentheri* shows strong site philopatry and high genetic structure due to its reliance on consistently moist habitats such as ephemeral creek lines (Cummins et al. 2019).

Suboptimal terrestrial habitat between discrete ponds may also influence dispersal and contribute to the genetic differentiation and sub‐structuring observed in the Watagans population. The Central Coast Ranges have experienced extensive logging and ongoing recreational use (e.g., 4WD touring and mountain biking), which can degrade vegetation structure and canopy cover and negatively impact amphibian movement (DECCW 2010; Graeter et al. 2008; Popescu and Hunter Jr 2011; Todd et al. 2009). Conversely, *R. littlejohni* has been observed to breed in ephemeral ponds formed by wheel ruts in off‐road areas, indicating some capacity to use novel, human‐modified habitats.

Human interventions in the natural landscape, specifically the creation of artificial ponds, may have significantly influenced the population structure of *R. littlejohni*. These ponds, initially built for purposes such as fire management, have inadvertently provided alternative breeding habitats for the species. As a result, individuals that would have typically bred in natural creek systems may be using these constructed ponds, potentially because they are more likely to retain water. However, it is also possible that frogs disperse more widely, but only those that locate suitable breeding sites, such as these ponds, successfully reproduce and persist in the population. Another explanation for *R. littlejohni's* affinity for constructed ponds could be an interaction with the amphibian fungal disease, chytridiomycosis (“chytrid”). Constructed ponds may offer better breeding habitats in the face of chytrid infection by providing micro‐climate conditions less suitable to chytrid's growth and transmissibility. Chytrid grows best between 17°C and 23°C, and both lab and field studies have shown a link between infection rates and environmental temperature, with higher infection loads occurring at cooler temperatures (Forrest and Schlaepfer 2011; Klop‐Toker, Valdez, et al. 2021; Piotrowski et al. 2004). Constructed ponds in the Central Coast Ranges are primarily located on ridgetops, which likely support warmer micro‐climates compared to natural creek lines in valleys that are likely to be cooler and shadier. Future studies are needed to investigate micro‐climate conditions and their influence on chytrid infection in this region to help understand the influence of disease on population structure.

### 4.2 Environmental Stochastic Impact on Genetic Diversity and Inbreeding

The projected increase in temperatures and drought conditions due to global warming raises significant concerns for vulnerable amphibian populations, particularly those with reduced genetic diversity that limits their adaptive ability in stochastic environments. Previous research on amphibians have shown that environmental stochasticity in metapopulations leads to higher dispersal rates and high fecundity but shorter lifespans. Whereas, in populations in more predictable environments, dispersal and fecundity was low and lifespan increased (Cayuela et al. 2016; Comins et al. 1980). Across our study period, the Central Coast Ranges population of *R. littlejohni* experienced unpredictable environments, including drought like conditions in 2020 and 2023, but also heavy rainfall associated with La Niña in 2022, the cool phase of the El Niño‐Southern Oscillation (Bureau of Meteorology 2023; CSIRO and Bureau of Meteorology 2022). We observed limited dispersal between the two sub‐populations during periods of drought, indicating that frogs remain at reliable water sources during these periods. We also observed evidence of dispersal after heavy rainfall, demonstrating that dispersal patterns changed in response to stochastic events. The provision of waterbodies with hydroperiods suitable to support the tadpole phase of *R. littlejohni* life cycle (3–9 months) with high connectivity in the landscape may facilitate gene flow and genetic mixing between sub‐populations. This suggests that hydrological conditions play a critical role in shaping population structure, with wetter periods promoting connectivity and drier periods reinforcing genetic separation. This trend has also been observed in other amphibian species, such as *Pelodytes punctatus* and *Hyla meridionalis* , where increased rainfall was associated with lower extinction rates and increased colonization at ponds for both species (Cayuela et al. 2012).

Populations with high inbreeding and low heterozygosity are more vulnerable to stochastic events due to their reduced adaptive potential and may be at increased risk of inbreeding depression (Frankham et al. 2019; Kyriazis et al. 2021). This may be relevant for the Central Coast Ranges *R. littlejohni* population, particularly the southern sub‐population, which exhibits increased inbreeding, higher mean kinship and lower heterozygosity. Reduced genetic diversity, combined with limited dispersal and low effective population size, may increase the risk of processes such as mutational meltdown, an extinction vortex driven by the accumulation of deleterious mutations (Hedrick 2009; Keller and Waller 2002; Lynch et al. 1995a, 1995b). These finding suggest that the southern sub‐population may be vulnerable to declines in genetic fitness and highlight the importances of monitoring its long‐term viability.

### 4.3 Conservation Recommendations

Translocation and head‐starting programmes may be an effective solution to improving the genetic status of this endangered tree frog. Head‐starting refers to the practice of collecting eggs or larvae from a breeding pond, raising them in a controlled environment to reduce mortality during vulnerable stages, and then releasing the individuals back into their point of capture. It is distinct from translocation, which involves moving individuals between isolated populations (IUCN/SSC 2013). However, this study highlights the importance of suitable climatic conditions as a key ingredient in maximizing the benefits of translocation and head‐starting programmes, by facilitating dispersal and thus improving the potential for outbreeding. Conducting these conservation actions during periods of extended rainfall may facilitate natural dispersal due to the species' inherent movement capacity observed in this and other studies (Stock et al. 2023). Under these circumstances, head‐starting alone may boost population size but risks exacerbating inbreeding and reducing genetic diversity if released individuals remain localized at the natal pond of release, thereby increasing the proportion of related individuals (Wang and Ryman 2001). Alternatively, translocating individuals from outside the population could introduce new genetic profiles into the system, improving the genetic diversity of the population regardless of dispersal ability.

Given that dispersal and gene flow are linked to improved genetic variability and decreased inbreeding (Frankham et al. 2019), we recommend the creation of additional waterbodies less than 200 m apart to encourage movement between the northern and southern sub‐populations. Frogs are likely to colonize nearby ponds first and use these as stepping stones to occupy more distant ponds, therefore increasing gene flow (Simberloff and Abele 1976). The addition of strategically placed breeding habitat could promote the formation of a large, functionally connected metapopulation across the Central Coast Ranges and increase reproductive output, contributing to high effective population size. However, in the southern sub‐population, increases in effective population size (*N*_e_) following breeding events were accompanied by high mean kinship, indicating that reproduction may be dominated by closely related individuals or that reproductive success is skewed (Potvin et al. 2017). Such patterns are unlikely to improve genetic diversity and may further exacerbate inbreeding. Therefore, habitat creation alone may be insufficient to improve genetic health. We propose that targeted translocation of juveniles from the northern sub‐population, or from other *R. littlejohni* populations, may help to increase genetic diversity and reduce inbreeding in the southern sub‐population. This is supported by the higher heterozygosity and lower mean kinship observed in the northern sub‐population, indicating its potential to act as a genetic source. Ongoing genetic monitoring will be essential to assess the effectiveness of these inventions and to evaluate long‐term changes in genetic diversity under varying environmental conditions.

Maintaining the integrity of first order streams remains a key action for securing the viability of this population of *R. littlejohni*. Our study provides some preliminary findings that artificial ponds may limit genetic mixing due to their isolated nature, potentially leading to higher genetic structuring and reduced gene flow between sub‐populations. Whereas stream systems provide natural connectivity, allowing for greater dispersal and genetic exchange between breeding sites. On the other hand, the creation of isolated ponds along warmer ridge‐lines may have been the key element in protecting this population from localized extinction in response to chytrid, which is likely to be more prevalent and transmissible in cool, creek lines. Therefore, this study system highlights how features that may promote breeding (i.e., constructed ponds), act in contrast to features that support dispersal (i.e., streams), and highlights the complexity of conserving species in the face of multiple threats such as disease, climate change, and modified landscapes. Protecting and maintaining natural stream habitats may help improve geneflow and lower inbreeding rates, while discrete ponds may offer productive breeding habitats that boost/maintain population size. Either way, restoring the long‐term viability of *R. littlejohni* likely depends on multiple conservation actions.

## 5 Conclusion

We found that intense rainfall events in cooler months had a positive effect on dispersal ability and reduced genetic sub‐structuring between *R. littlejohni* populations, whereas drought conditions appeared to limit dispersal. In areas where both natural creek lines and constructed ponds served as breeding habitats, we observed increased heterozygosity and decreased kinship compared to areas with only constructed ponds. In the southern sub‐population, where *R. littlejohni* relied significantly on constructed ponds as breeding habitat, inbreeding steadily increased over the years, even during years of high rainfall. This highlights that facilitating dispersal and ensuring habitat continuity between ponds are key to improving the genetic health and recovery of the Central Coast Ranges *R. littlejohni* population. Without dispersal and subsequent gene flow between the two sub‐populations to enhance genetic variability, this already small population risks entering an extinction vortex, where reduced genetic diversity leads to lower fitness, decreased reproduction, and higher mortality, perpetuating population decline (Fagan and Holmes 2006). To halt this cycle and support population recovery, further conservation intervention is necessary. Other populations, like the small, isolated Blue Mountains population of *R. littlejohni*, are also likely to exhibit similar genetic structuring as they also predominantly rely on constructed ponds for breeding. These populations may benefit from translocation initiatives and enhanced pond connectivity. Given the potential for genetic rescue, managing the species as a metapopulation could facilitate gene flow and maximize genetic diversity among populations. Moreover, species exhibiting genetic sub‐structuring and low diversity, such as the critically endangered *Litoria myola* (Bertola et al. 2023), could similarly benefit from targeted connectivity initiatives and population reinforcement strategies to enhance gene flow and reduce the risks of inbreeding depression.

## Author Contributions

**Nadine Nolan:** conceptualization (equal). **Sarah Stock:** conceptualization (equal). **Alex Callen:** conceptualization (equal). **Matt W. Hayward:** conceptualization (equal). **Sam Wallace:** conceptualization (equal). **Rose Upton:** conceptualization (equal). **Michael Mahony:** conceptualization (equal). **Kaya Klop‐Toker:** conceptualization (equal).

## Funding

This work was supported by the Australian Society of Herpetologists, and the Commonwealth Bushfire Recovery Program which is undertaken in collaboration with the NSW Government through the Saving Our Species Program, which is administered by the Environment and Energy Services (EES), Department of Planning, Industry, and Environment.

## Disclosure

Benefits Generated: Benefits from this research accrue from the sharing of our data and results on public databases as described above.

## Conflicts of Interest

The authors declare no conflicts of interest.

## Supporting information


**Table S1:** Recapture dates, ponds and distance moved of *Litoria littlejohni* in the Central Coast Ranges population, identified by microchips and SNP genotyping.
**Figure S1:** Genomic relationship matrix (GRM) based on genetic samples collected from *L. littlejohni* across 4 years within the Central Coast Ranges population, including two known siblings. Each node represents an individual frog. Colour‐coded nodes represent the 12 sample sites and connecting lines show related individuals. Line colours represent the level of kinship based the *gl.grm* function. Kinship values range from 0 to 1, with values from the same individual/twin represented by 1 and unrelated individuals represented by 0. In‐between values represent various levels of relatedness. Strength of node colour indicates the number of other relationship that node is connected to.
**Figure S2:** Determination of the Optimal Number of Genetic Clusters (*K*) for Dataset 1 Using the Puechmaille Method.6/05/2026.
**Figure S3:** Structure results for Dataset 1 *K* = 1–2 created using Structure Selector on the Central Coast Range *L. littlejohni* population. Northern sub‐population = pop_1 and southern sub‐populations = pop_2.
**Figure S4:** Bayesian clustering and model‐based inference of genetic structure in the Central Coast population of *Litoria littlejohni* : Delta *K* and likelihood‐based approaches.
**Table S2:** Genetic diversity metrics for *Litoria littlejohni* northern and southern sub‐populations in the Central Coast ranges population.
**Table S3:** Pairwise comparisons of autosomal heterozygosity between for *Litoria littlejohni* .
**Table S4:** Pairwise comparisons of inbreeding (*F*) between year and sub‐populations of *Litoria littlejohni* .

## Data Availability

Raw sequence reads are deposited in Figshare under DOI: https://doi.org/10.6084/m9.figshare.30205849. Processed SNP genotype data and associated specimen metadata are provided as Supporting Information files within the same repository record.
