# Supplementary material for: Assessing Genetic Diversity and Inbreeding Effects in an Endangered Amphibian: Effects of Ecological Restoration and Stochasticity
Source: Ecol Evol. 2026 May 22;16(5):e73649. doi: 10.1002/ece3.73649 (PMC13240253; doi:10.1002/ece3.73649)
Supplement: Supplementary file 1 — Table S1: Recapture dates, ponds and distance moved of Litoria littlejohni in the Central Coast Ranges population, identified by microchips and SNP genotyping. Figure S1: Genomic relationship matrix (GRM) based on genetic samples collected from L. littlejohni across 4 years within the Central Coast Ranges population, including two known siblings. Each node represents an individual frog. Colour‐coded nodes represent the 12 sample sites and connecting lines show related individuals. Line colours represent the level of kinship based the gl.grm function. Kinship values range from 0 to 1, with values from the same individual/twin represented by 1 and unrelated individuals represented by 0. In‐between values represent various levels of relatedness. Strength of node colour indicates the number of other relationship that node is connected to. Figure S2: Determination of the Optimal Number of Genetic Clusters (K) for Dataset 1 Using the Puechmaille Method.6/05/2026. Figure S3: Structure results for Dataset 1 K = 1–2 created using Structure Selector on the Central Coast Range L. littlejohni population. Northern sub‐population = pop_1 and southern sub‐populations = pop_2. Figure S4: Bayesian clustering and model‐based inference of genetic structure in the Central Coast population of Litoria littlejohni : Delta K and likelihood‐based approaches. Table S2: Genetic diversity metrics for Litoria littlejohni northern and southern sub‐populations in the Central Coast ranges population. Table S3: Pairwise comparisons of autosomal heterozygosity between for Litoria littlejohni . Table S4: Pairwise comparisons of inbreeding (F) between year and sub‐populations of Litoria littlejohni . [file ECE3-16-e73649-s001.docx]

**Assessing Genetic Diversity and Inbreeding Effects in an Endangered Amphibian: Effects of Ecological Restoration and Stochasticity**

**Nadine Nolan**^1^, Sarah Stock^1^, Alex Callen^1^, Matt W. Hayward^1,2^, Sam Wallace^1^, Rose Upton^1^, Michael Mahony^1^ and Kaya Klop-Toker^1^

**Supplementary Materials:**

**Table S1:** Recapture dates, ponds and distance moved of *Litoria littlejohni* in the Central Coast Ranges population, identified by microchips and SNP genotyping.

**Animal First Capture First Capture Second Capture Second Capture Distance**

**Date Pond Date Pond Moved (m)**

**Microchip Recapture**

| Frog 1 | 2022 | Pines Rd 1 | 2022 | Pines Picnic |  | 170 |
| --- | --- | --- | --- | --- | --- | --- |
| Frog 2 | 2022 | OTP | 2022 | OTP |  | 0 |
| Frog 3 | 2022 | OTP | 2022 | OTP |  | 0 |
| Frog 4 | 2022 | Kcas2 | 2022 | Kcas2 |  | 0 |
| Frog 5 | 2022 | Fawcetts | 2022 | Fawcetts |  | 0 |
| Frog 6 | 2022 | Sawmill B | 2022 | Sawmill A |  | 180 |
| Frog 7 | 2023 | OTP | 2023 | Pines Picnic |  | 680 |
| Frog 8 | 2023 | OTP | 2023 | OTP |  | 0 |
| Frog 9 | 2023 | OTP | 2023 | OTP |  | 0 |
| Frog 10 | 2023 | OTP | 2023 | OTP |  | 0 |
| Frog 11 | 2022 | Sawmill B | 2022 | Elbow |  | 180 |
| Frog 12 | 2023 | Sawmill B | 2023 | Sawmill B |  | 0 |

**SNP Recapture**

| Frog 13 | 2020 | Fawcetts | 2020 | Fawcetts |  | 0 |
| --- | --- | --- | --- | --- | --- | --- |
| Frog 14 | 2020 | Lost Pond | 2020 | Lost Pond |  | 0 |
| Frog 15 | 2021 | Fawcetts | 2023 | Fawcetts |  | 0 |
| Frog 16 | 2021 | Fawcetts | 2021 | Fawcetts |  | 0 |
| Frog 17 | 2021 | Fawcetts | 2022 | Fawcetts |  | 0 |


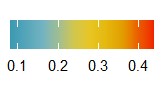

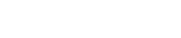


Relatedness


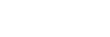


Pond


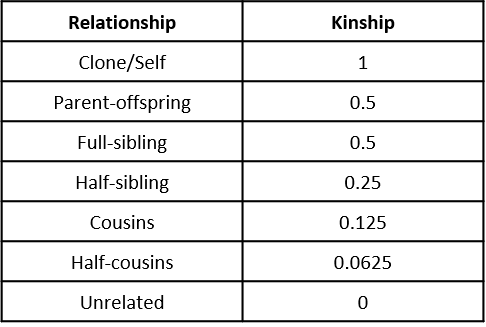

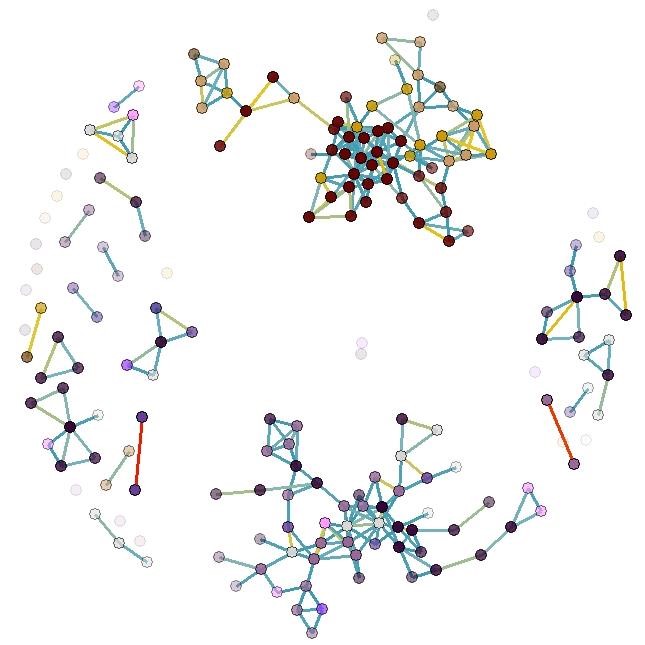

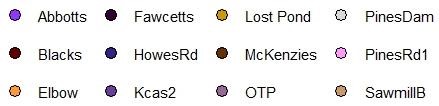


**Fig. S1.** Genomic relationship matrix (GRM) based on genetic samples collected from *L. littlejohni* across four years within the Central Coast Ranges population, including two known siblings. Each node represents an individual frog. Color-coded nodes represent the 12 sample sites and

connecting lines show related individuals. Line colors represent the level of kinship based the

*gl.grm* function. Kinship values range from 0-1, with values from the same individual/twin

represented by 1 and unrelated individuals represented by 0. In-between values represent various levels of relatedness. Strength of node color indicates the number of other relationship that node is connected to.


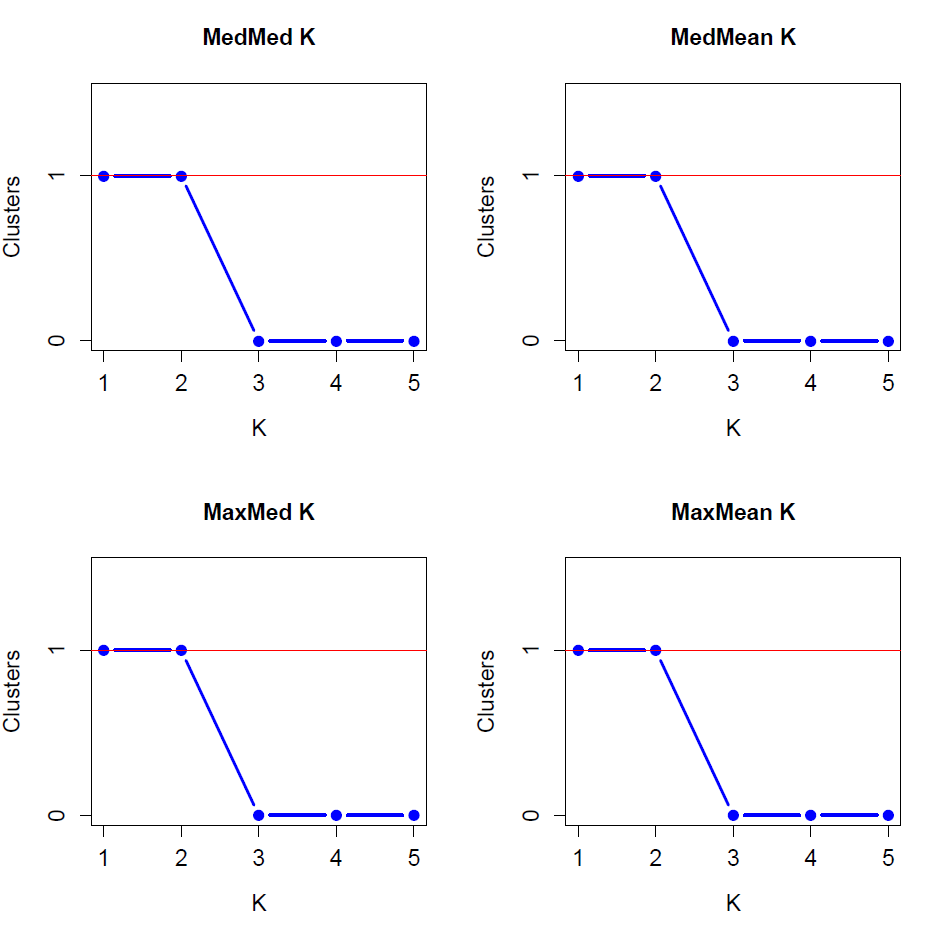


**Fig. S2.** Determination of the Optimal Number of Genetic Clusters (K) for Dataset 1 Using the Puechmaille Method.26/09/2025


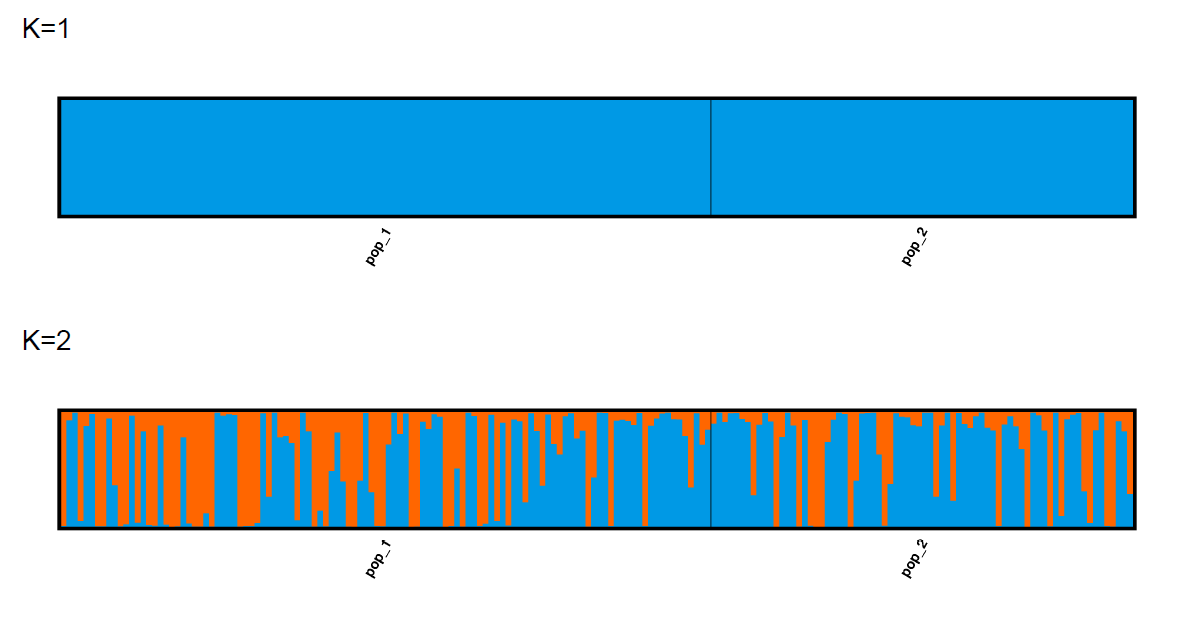


**Fig. S3.** Structure results for Dataset 1 K = 1-2 created using

Structure Selector on the Central Coast Range *L. littlejohni* population. Northern sub-population = pop_1 and southern sub-populations = pop_2.


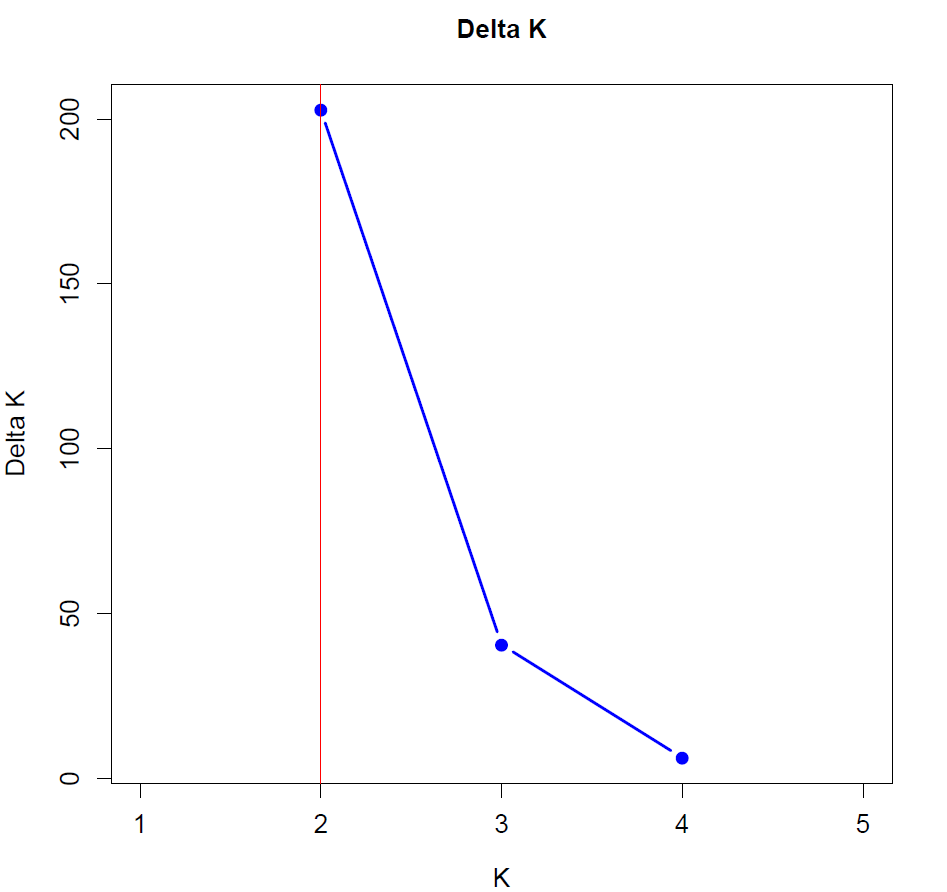

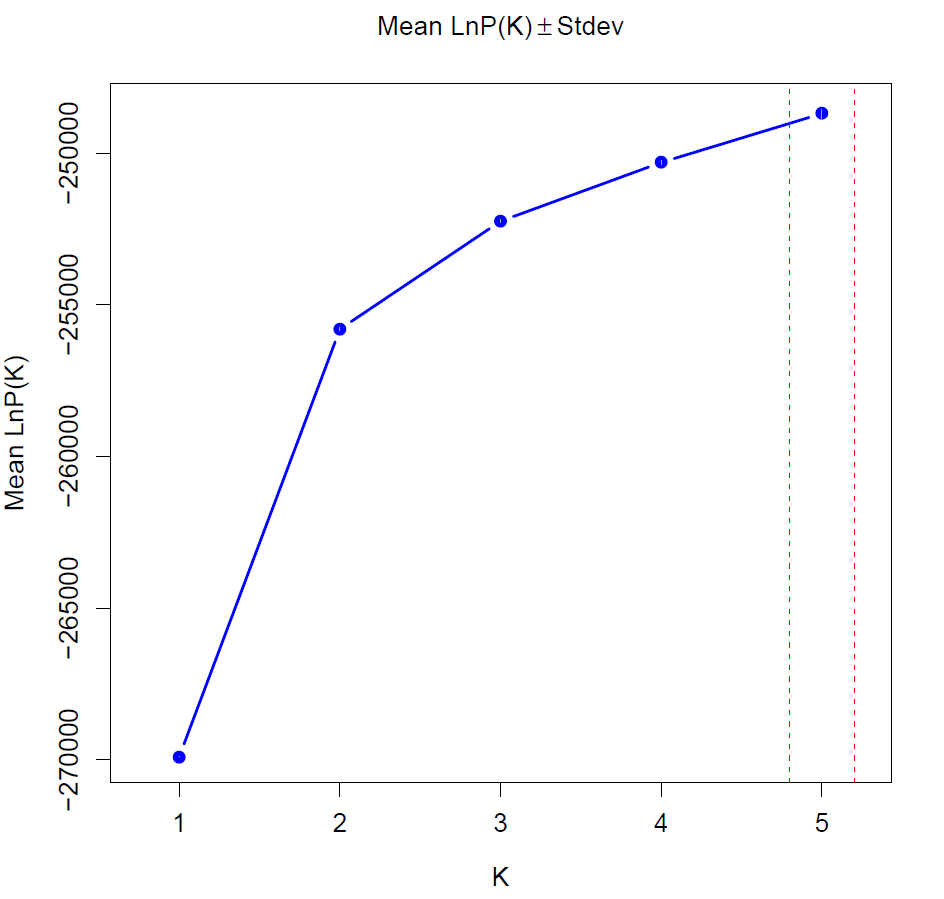


**Fig. S4.** Bayesian Clustering and Model-Based Inference of Genetic Structure in the Central Coast Population of Litoria littlejohni: Delta K and Likelihood-Based Approaches.

**Table S2:** Genetic Diversity metrics for *Litoria littlejohni* northern and southern sub-populations in

the Central Coast Ranges population

**Population North Subpopulation South Subpopulation**

| Year | 2020 | 2021 | 2022 | 2023 | 2020 | 2021 | 2022 | 2023 |
| --- | --- | --- | --- | --- | --- | --- | --- | --- |
| **Individual** | 13 | 41 | 25 | 38 | 18 | 29 | 14 | 9 |
| **Loci** | 193 | 1983 | 1781 | 1588 | 193 | 1983 | 1781 | 1588 |
| **HO (SD)** | 0.25  (0.20) | 0.23  (0.18) | 0.26  (0.19) | 0.3  (0.16) | 0.24  (0.22) | 0.23  (0.19) | 0.25  (0.19) | 0.3  (0.22) |
| **HE (SD)** | 0.25  (0.18) | 0.25  (0.18) | 0.26  (0.17) | 0.3  (0.15) | 0.22  (0.18) | 0.24  (0.19) | 0.26  (0.18) | 0.28  (0.18) |
| **HE-U (SD)** | 0.26  0.18) | 0.25  (0.18) | 0.27  (0.18) | 0.31  (0.15) | 0.23  (0.19) | 0.24  (0.19) | 0.26  (0.19) | 0.3  (0.2) |
| **FIS (CIs)** | 0.01  (0.05,  0.07) | 0.06  (0.05,  0.07) | 0.04  (0.03,  0.05) | 0.05  (0.04,  0.07) | -0.03  (-0.1,  0.02) | 0.06  (0.05,  0.07) | 0.04  (0.03,  0.06) | 0.03  (0.01  0.05) |
| **F** | 0.04  (0.08,  0.17) | 0.07  (0.02,  0.12) | 0.04  (-0.01,  0.09) | 0.05  (0.001,  0.11) | -0.001  (-0.13,  0.15) | 0.11  (0.05,  0.15) | 0.08  (0.03,  0.14) | 0.16  (0.1,  0.22) |
| **Mean**  **Kinship** | 0.01  (0.19,  0.19) | 0.05  (-0.01,  0.11) | 0.03  (-0.04,  0.08) | 0.02  -(0.04,  0.08) | 0.09  (-0.10,  0.28) | 0.11  (0.05,  0.16) | 0.09  (0.04,  0.16) | 0.21  (0.13,  0.25) |
| **NE (CIs)** | 29.1  (21.5,  43.2) | 23.5  (23.3,  23.8) | 31.5  (30.9,  32.1) | 34.7  (34.3,  35.1) | 28.0  (22.0,  37.4) | 26.1  (25.8,  26.5) | 18.8  (18.4,  19.3) | 51.2  (47.0,  56.2) |

The number of individuals within each subpopulation followed by the number of loci, Observed Heterozygosity (Ho), Expected Hardy-Weinberg Heterozygosity (He), Unbiased expected heterozygosity (HE-U), inbreeding coefficient (FIS), lynchrd co-efficient of inbreeding identity by descent method (F), Mean Kinship (quellergt), and Effective population size (NE). Values in brackets indicate standard deviation (SD) and 95% confidence interval (CIs).

**Table S3**: Pairwise comparisons of autosomal heterozygosity between for *Litoria littlejohni*

| **Contrast**  **Year - Year** |  | **Estimate** | **SE** | **T ratio** | **P value** |
| --- | --- | --- | --- | --- | --- |
| 2020 - 2021 |  | 0.0037 | 0.0011 | 3.276 | *0.0068 |
| 2020 - 2022 |  | 0.0006 | 0.0012 | 0.476 | 0.9641 |
| 2020 - 2023 |  | 0.002 | 0.0012 | 1.850 | 0.2534 |
| 2021 - 2022 |  | -0.003 | 0.0010 | -2.98 | *0.0168 |
| 2021 - 2023 |  | -0.001 | 0.0010 | -1.42 | 0.4850 |
| 2022 - 2023 |  | 0.0017 | 0.0011 | 1.493 | 0.4432 |

- Significant pairwise comparisons

**Table S4**: Pairwise comparisons of inbreeding (F) between year and subpopulations of *Litoria littlejohni*

| **Contrast**  **Year – Sub-population** | **Estimate** | **SE** | **T ratio** | **P value** |
| --- | --- | --- | --- | --- |
| 2020 North - 2021 North | -0.0352 | 0.024 | -1.467 | 0.8237 |
| 2020 North - 2022 North | -0.0025 | 0.026 | -0.099 | 1.0 |
| 2020 North - 2023 North | -0.0161 | 0.0242 | -0.665 | 0.9978 |
| 2020 North - 2020 South | 0.0342 | 0.0278 | 1.232 | 0.9212 |
| 2020 North - 2021 South | -0.0587 | 0.0252 | -2.333 | 0.2818 |
| 2020 North - 2022 South | -0.0485 | 0.0291 | -1.669 | 0.7069 |
| 2020 North - 2023 South | -0.1246 | 0.0327 | -3.809 | *0.0047 |
| 2021 North - 2022 North | 0.0326 | 0.0194 | 1.685 | 0.697 |
| 2021 North - 2023 North | 0.0191 | 0.017 | 1.125 | 0.9505 |
| 2021 North - 2020 South | 0.0695 | 0.0218 | 3.193 | 0.0348 |
| 2021 North - 2021 South | -0.0235 | 0.0183 | -1.284 | 0.9036 |
| 2021 North - 2022 South | -0.0132 | 0.0234 | -0.568 | 0.9992 |
| 2021 North - 2023 South | -0.0893 | 0.0278 | -3.218 | *0.0324 |
| 2022 North - 2023 North | -0.0135 | 0.0197 | -0.689 | 0.9972 |
| 2022 North - 2020 South | 0.0368 | 0.0239 | 1.54 | 0.7849 |
| 2022 North - 2021 South | -0.0562 | 0.0208 | -2.699 | 0.1299 |
| 2022 North - 2022 South | -0.0459 | 0.0254 | -1.811 | 0.6136 |
| 2022 North - 2023 South | -0.1220 | 0.0295 | -4.138 | *0.0014 |
| 2023 North - 2020 South | 0.0504 | 0.022 | 2.289 | 0.3058 |
| 2023 North - 2021 South | -0.0426 | 0.0186 | -2.291 | 0.3043 |
| 2023 North - 2022 South | -0.0323 | 0.0236 | -1.373 | 0.8683 |
| 2023 North - 2023 South | -0.1085 | 0.028 | -3.879 | *0.0036 |
| 2020 South - 2021 South | -0.0930 | 0.0231 | -4.036 | *0.002 |
| 2020 South - 2022 South | -0.0827 | 0.0272 | -3.04 | 0.054 |
| 2020 South - 2023 South | -0.1589 | 0.0311 | -5.108 | *0.0001 |
| 2021 South - 2022 South | 0.01024 | 0.0246 | 0.417 | 0.9999 |
| 2021 South - 2023 South | -0.0658 | 0.0288 | -2.288 | 0.3063 |
| 2022 South - 2023 South | -0.0761 | 0.0322 | -2.361 | 0.2675 |

- Significant pairwise comparisons
